# A randomized phase II study of carboplatin plus pegylated liposomal doxorubicin versus carboplatin plus paclitaxel in platinum sensitive ovarian cancer patients: a Hellenic Cooperative Oncology Group study

**DOI:** 10.1186/1741-7015-8-3

**Published:** 2010-01-07

**Authors:** Dimitrios Bafaloukos, Helena Linardou, Gerasimos Aravantinos, Christos Papadimitriou, Aristotelis Bamias, George Fountzilas, Haralabos P Kalofonos, Paris Kosmidis, Eleni Timotheadou, Thomas Makatsoris, Epaminondas Samantas, Evangelos Briasoulis, Christos Christodoulou, Pavlos Papakostas, Dimitrios Pectasides, Athanasios M Dimopoulos

**Affiliations:** 1 1st Oncology Department, Metropolitan Hospital, Athens, Greece; 2 3rd Oncology Clinic, Agii Anargiri Cancer Hospital, Athens, Greece; 3 Department of Clinical Therapeutics, Alexandra Hospital, University of Athens School of Medicine, Athens, Greece; 4 Department of Oncology, Papageorgiou Hospital, Aristotle University of Thessaloniki School of Medicine, Thessaloniki, Greece; 5 Department of Oncology University of Patras Medical School, Patras, Greece; 6 2nd Oncology Department, Hygeia Hospital, Athens, Greece; 7 Department of Oncology, Ioannina University Hospital, Ioannina, Greece; 8 2nd Oncology Department, Metropolitan Hospital, Athens, Greece; 9 Department of Oncology, Ippokration Hospital, Athens, Greece; 10 Department of Oncology, University General Hospital Attikon, Athens, Greece

## Abstract

**Background:**

Platinum-based combinations are the standard second-line treatment for platinum-sensitive ovarian cancer (OC). This randomized phase II study was undertaken in order to compare the combination of carboplatin and pegylated liposomal doxorubicin (LD) with carboplatin and paclitaxel (CP) in this setting.

**Methods:**

Patients with histologically confirmed recurrent OC, at the time of or more than 6 months after platinum-based chemotherapy, were randomized to six cycles of CP (carboplatin AUC5 + paclitaxel 175 mg/m^2^, d1q21) or CLD (carboplatin AUC5 + pegylated LD 45 mg/m^2^, d1q28).

**Results:**

A total of 189 eligible patients (CP 96, CLD 93), with a median age of 63 years, median Performance Status (PS) 0 and a median platinum free interval (PFI) of 16.5 months, entered the study. Discontinuation due to toxicity was higher in the CP patients (13.5% versus 3%, *P *= 0.016). The overall response rate was similar: CP 58% versus CLD 51%, *P *= 0.309 (Complete Response; CR 34% versus 23%) and there was no statistical difference in time-to-progression (TTP) or overall survival (OS; TTP 10.8 months CP versus 11.8 CLD, *P *= 0.904; OS 29.4 months CP versus 24.7 CLD, *P *= 0.454). No toxic deaths were recorded. Neutropenia was the most commonly seen severe toxicity (CP 30% versus CLD 35%). More frequent in CLD were severe thrombocytopenia (11% versus 2%, *P *= 0.016), skin toxicity and Palmar-plantar erythrodysesthesia (PPE) grade 1-2 (38% versus 9%, *P*< 0.001), while grade 3 neurotoxicity and alopecia were higher in CP (7% versus 0%, *P *= 0.029, 20% versus 5%, *P *= 0.003). PS and PFI were independent prognostic factors for TTP and OS.

**Conclusions:**

The combination of pegylated LD with carboplatin is effective, showing less neurotoxicity and alopecia than paclitaxel-carboplatin. It thus warrants a further phase III evaluation as an alternative treatment option for platinum-sensitive OC patients.

**Trial Registration:**

Australian New Zealand Clinical Trials Registry: ACTRN12609000436279

## Background

Ovarian cancer (OC) remains the leading cause of death due to gynaecological malignancies [1]. The standard first-line treatment is a platinum-paclitaxel combination, achieving complete response rates of up to 50%. However, despite progress in first-line therapy, more than 60% of patients relapse and die from chemoresistant disease [2-4]. Therefore, the majority of patients will become candidates for second-line treatment. It is generally accepted that a response to platinum and the platinum-free interval (PFI) of relapse, are the determining clinical surrogates for response and prognosis of recurrent OC [5,6]. Platinum-sensitive disease is generally defined as disease relapsing ≥ 12 months post previous platinum-based chemotherapy, while relapse within 6-12 months is considered to be partially platinum-sensitive For patients with platinum-sensitivity re-treatment with platinum is considered to be the preferred option with an overall probability of response of around 30% [7,8].

Recent randomized phase III studies have shown that combinations of platinum with paclitaxel offer significant improvements over platinum monotherapies for platinum-sensitive and partially-sensitive disease. This applies to both progression-free survival (PFS) and overall survival (OS), and was demonstrated in a large patient population (802 patients) who participated in the ICON4/AGO-OVAR trial [9]. Most patients had a PFI > 12 months and more than half had received single-agent platinum as first-line therapy. Neurotoxicity was the most important limiting adverse event, with 20% of patients experiencing grade 2-4 neurotoxicity in the paclitaxel-platinum arm compared to 1% with platinum monotherapy. Following this, as well as other randomized phase II studies [10], the combination of paclitaxel plus platinum (preferably carboplatin) has emerged as the standard of care for patients with platinum-sensitive OC. However, as neurotoxicity could be a significant and persistent problem, re-treatment with the same regimen may not be feasible because of the cumulative neurotoxicity of both platinum and paclitaxel. Several groups are addressing this issue, comparing platinum-based doublets to platinum. The recent AGO-OVAR 2.5 trial evaluated a carboplatin-gemcitabine combination over carboplatin in 356 relapsed OC patients with a PFI of ≥ 6 months, showing that the combination significantly improves PFS and responses without worsening quality-of-life (QoL) [11].

Other agents with single-agent efficacy in second-line relapsed OC include pegylated liposomal doxorubicin (LD) and topotecan. Pegylated LD is effective as a monotherapy in platinum-sensitive and platinum-resistant relapse and shows a similar efficacy to paclitaxel but with a different toxicity profile [12]. Pegylated LD was superior to topotecan for PFS and OS with a more favourable profile in terms of tolerance, cost and QoL [13]. In recent phase II studies, pegylated LD has demonstrated significant activity (response rate [RR] 56%) combined with carboplatin [14-16]. Pegylated LD monotherapy is recommended for platinum-resistant and partially-platinum sensitive OC patients [7].

The above form the rationale for this randomized phase II study, which is designed to evaluate the combination of carboplatin plus pegylated LD and the standard carboplatin plus paclitaxel, in patients with OC relapsing at least 6 months after first-line platinum-based therapy.

## Methods

### Eligibility criteria

Women over 18 years old, with a histologically confirmed recurrent OC, ≥ 6 months after platinum-based chemotherapy, entered this study. Patients with bidimensionally measurable disease or only elevated serum CA-125 (≥ twice the upper limit of normal), with Eastern Cooperative Oncology Group performance status (PS) 0-2 and life expectancy of ≥ 3 months were eligible. Adequate bone marrow, hepatic and renal functions were required. Patients with a history of malignancy, other than a completely excised *in situ *carcinoma of the cervix or basal carcinoma of the skin, prior or recurrent central nervous system metastases, serious cardiac disease, other serious medical illness or an inability to comply with the treatment plan and follow-up visits were excluded. Also patients with residual neurotoxicity from previous platinum and/or taxane chemotherapy were excluded. The clinical protocol and collateral translational research studies were approved by the HeCOG Protocol Review Committee and by the institutional review boards in participating institutions. Written informed consent was obtained from all patients before study entry.

### Treatment and dose modifications

Pre-treatment evaluation included a medical history, physical examination, chest X-ray and abdominal computed tomography scan, electrocardiogram, complete blood count (CBC), biochemistry and CA-125 determination. In addition, a baseline neurological examination was performed and was recommended at every cycle.

**Figure 1:**
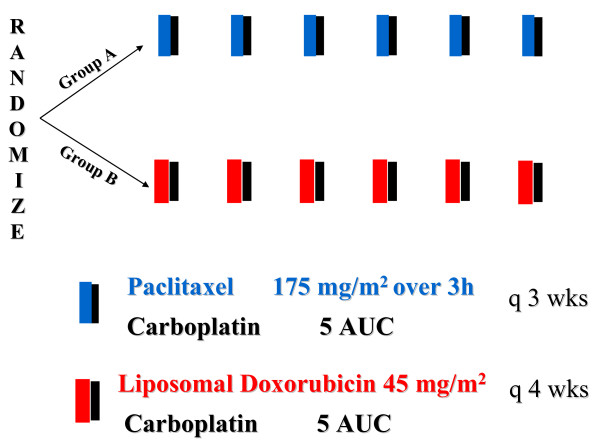
**Treatment schema**.

Randomization was performed at the central HeCOG Data Office in Athens. No stratification criteria were applied at randomisation. The chemotherapy regimens are depicted in Figure 1. Patients in the control arm (A) received paclitaxel at 175 mg/m^2 ^as a 3 h infusion followed by carboplatin at an area under the curve (AUC) 5, on day 1. Cycles were repeated every 21 days. Patients in the experimental arm (B) received pegylated liposomal doxorubicin at 45 mg/m^2 ^followed by carboplatin at AUC 5, on day 1. Cycles were repeated every 28 days. The dose of carboplatin based on AUC was calculated by the estimated creatinine clearance using Calvert's formula [17]. All patients received standard premedication of dexamethasone, dyphenhydramine and ranitidine prior to paclitaxel, orally 12 h prior to paclitaxel administration and again intravenously 30 min prior to paclitaxel infusion. The same premedication was administered intravenously only prior to Pegylated LD infusion for the patients in the carboplatin plus pegylated LD (CLD) arm. Six cycles of chemotherapy were administered, unless evidence of disease progression or unacceptable toxicity occurred.

CBC and biochemistry were carried out before each cycle. CBC was performed routinely only on day1 and was repeated during cycles only in cases of fever > 38°C, severe stomatitis or diarrhoea. Chemotherapy was administered if absolute neutrophil count (ANC) ≥1.5 × 10^9^/L and platelet count ≥ 100 × 10^9^/L. If ANC was < 1.5 × 10^9^/L on day 1, a granulocyte colony-stimulating factor (G-CSF) was used to speed recovery. A maximum of 2 weeks delay was allowed for toxicity and treatment was discontinued if longer toxicity-related delays occurred. In cases of prolonged neutropenia (> 7 days with ANC < 0.5 × 10^9^/L) despite G-CSF use or febrile neutropenia, a 25% dose reduction for all drugs was applied additionally to G-CSF. For grade 3 and 4 thrombocytopenia, a 25% and a 50% dose reduction, respectively, was recommended for all drugs. If creatinine clearance was calculated as < 30 ml/min, treatment was delayed for a maximum of 2 weeks until recovery; otherwise the patient was withdrawn from the study. For cardiac arrhythmia, grade 3 hypersensitivity reactions and any non-haematological toxicity grade > 2 treatment was discontinued. Specifically, for grade 2 Palmar-plantar erythrodysesthesia (PPE), treatment was delayed for a maximum of 2 weeks until recovery to Grade 0 or 1. Toxicity criteria were those adopted by the World Health Organization (WHO). Tumour assessments for response were performed every two cycles. Patients receiving at least two cycles of chemotherapy were considered assessable for a response. Patients receiving at least one cycle of chemotherapy were assessable for toxicity. Standard WHO criteria were applied for an assessment of the response, as these were commonly used prior to the broad introduction of RECIST (response evaluation criteria in solid tumours) by our group and others, and at the time when this study was initiated. For patients without measurable disease, the response was determined based on repetitive CA-125 measurements using the algorithm proposed by Rustin *et al*. and according to CA-125 Rustin's criteria [18].

### Statistical analysis

This was a randomized phase II study. The principal endpoint was to evaluate the RR and toxicity of the two treatment regimens. Secondary endpoints were time-to-progression (TTP) and OS. The study was performed on the 'intent to treat' basis; thus all eligible patients were included in the analysis. The sample size was calculated on the assumption that a 20% difference in response rate to a baseline of 50% existed between the two groups. For an alpha and beta error of 0.05 and 0.20, respectively, 201 patients were needed. Exact binomial confidence intervals (CI) were used to determine the 95% upper and lower confidence limits of the response rate. OS was estimated from the initiation of treatment to the date of last follow-up or until the patient's death. TTP was calculated from the initiation of treatment to the first disease progression. In the calculation of TTP, deaths due to disease without previous documentation of progression were considered as events. The Kaplan-Meier method was used to estimate the time-to-event distributions. In order to account for potentially important prognostic factors, Cox regression analysis was performed for OS and TTP. Performance status (1, 2 versus 0), evidence of disease at study entry (measurable disease versus elevated marker), age (> 65 years versus ≤ 65 years), taxane-containing previous chemotherapy (yes versus no), PFI (≤ 12 months versus > 12 months) and randomization group were included in the models. The backwards selection procedure, using the maximum likelihood procedure, was used to conclude on the independent prognostic factors for OS and TTP. Categorical variables were compared using Fisher's exact test, while continuous variables were compared using the Mann-Whitney test. Patient information was collected on standard HeCOG study forms by authorized HeCOG Data Managers and entered in the HeCOG Database. The trial was monitored by certified HeCOG personnel.

## Results

### Patients' characteristics

**Figure 2:**
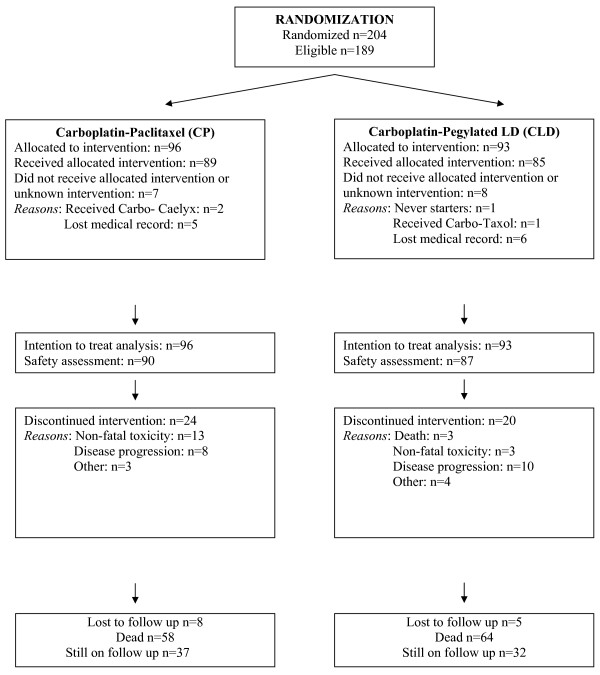
**Progress through the various stages of the trial**.

From October 1999 until December 2005, 204 patients were randomized. Fifteen patients were found to be non-eligible. The reasons for non-eligibility were: non-measurable disease (ascites only) without CA125 elevation (five patients); other cancer (five); no prior platinum-based chemotherapy (two); no evidence of disease at trial initiation (two); and in one case chemotherapy initiation 20 weeks prior randomization. Data from 189 eligible patients are presented (CP 96, CLD 93). Patients' characteristics are summarized in Table 1. Most patients had Performance Status (PS) = 0 (65% in CP, 59% in CLD). Primary disease characteristics, such as stage at initial diagnosis, histologic type and grade were balanced between both groups. Most patients had received only one previous line of chemotherapy (96% in both groups), usually containing a taxane (88% in CP, 93% in CLD). Most patients had a measurable disease, with 7% in CP and 10% in CLD having elevated CA-125 (≥ twice the upper limit of normal) as the only evidence of disease. Most patients had a platinum-free interval (PFI) of > 12 months (57% in CP, 72% in CLD), with a median PFI of 14.8 months in CP and 17.3 months in CLD. Although these values for PFI seemed more favourable in the CLD group, they did not differ significantly between the two groups (*P *= 0.191). The progress of patients through the various trial stages is shown in our flow diagram (Figure 2) according to the Consolidated Standards of Reporting Trials [19].

**Table 1:** Selected patient and tumour characteristics.

	Total *N *= 189	Group A (CP) *N *= 96	Group B (CLD) *N *= 93
**Age (years)**			

Median (range)	63 (37-89)	63 (37-81)	62 (38-89)

**Performance status**			

0	117 (62)	62 (65)	55 (59)

1	57 (30)	27 (28)	30 (32)

2	1 (1)	0 (0)	1 (1)

Unknown	14 (7)	7 (7)	7 (8)

**Stage at diagnosis**			

I	14 (7)	9 (9)	5 (5)

II	16 (9)	9 (9)	7 (8)

III	118 (62)	56 (58)	62 (67)

IV	28 (15)	15 (16)	13 (14)

Unknown	13 (7)	7 (7)	6 (7)

**Histologic grade**			

I	13 (7)	8 (8)	5 (5)

II	57 (30)	27 (28)	30 (32)

III	92 (49)	48 (50)	44 (47)

IV	3 (2)	1 (1)	2 (2)

Unknown	24 (13)	12 (13)	12 (13)

**Histologic type**			

Serous	143 (76)	71 (74)	72 (77)

Mucinous	3 (2)	0 (0)	3 (3)

Endometroid	13 (7)	6 (6)	7 (8)

Clear cell	6 (3)	3 (3)	3 (3)

Other	14 (7)	9 (9)	5 (5)

Unknown	10 (5)	7 (7)	3 (3)

**Prior therapy**			

Surgery	161 (85)	85 (89)	76 (82)

Taxane containing therapy	170 (90)	84 (88)	86 (93)

**Number of prior chemotherapy lines**			

1	181 (96)	92 (96)	89 (96)

> = 2	8 (4)	4 (4)	4 (4)

**Sites of disease**			

Abdominal	95 (50)	49 (51)	46 (50)

Abdominal and pleural effusion or ascites	43 (23)	18 (19)	25 (27)

Extra-abdominal	1 (1)	1 (1)	0 (0)

Both	9 (5)	5 (5)	4 (4)

Ascites or pleural effusion and elevated CA125	14 (7)	10 (10)	4 (4)

Elevated CA 125 as the only evidence of disease	16 (8)	7 (7)	9 (10)

Unknown	11 (6)	6 (6)	5 (5)

**Platinum free interval**			

6-12 months	54 (29)	32 (33)	22 (23)

12.1-24 months	70 (37)	32 (33)	38 (41)

> 24 months	52 (28)	23 (24)	29 (31)

Unknown	13 (7)	9 (9)	4 (4)

**Median platinum-free interval (months)**	16.5 (6-119)	14.8 (6-96)	17.3 (6-119)

**CA 125 at baseline**			

Median (range)	207 (4-6000)	210 (7-6000)	199 (4-3400)

CP, carboplatin plus paclitaxel; CLD, carboplatin plus pegylated liposomal doxorubicin

### Treatment characteristics

A total of 503 cycles of CP (median 6, range 1-9) and a total of 475 cycles of CLD (median 6, range 1-8) were administered. The median relative dose intensity for paclitaxel was 0.96 (range 0.66-1.07) and 0.92 (range 0.52-1.09) for pegylated LD, while the median cumulative dose of carboplatin was 2555 (range 370-4800) for CP and 2400 (range 270-5600) for CLD. Most patients in both groups completed the planned treatment (68% in CP and 70% in CLD). The rate of discontinuation due to toxicity was statistically significantly higher in the paclitaxel group (13.5% in CP versus 3% in CLD, *P *= 0.020). Other reasons for non-completion did not differ significantly between the two groups (CP versus CLD), and included: disease progression (8% versus 11%); tumour death (0% versus 3%); voluntary withdrawal (1% versus 0%); and doctor's decision (1% versus 1%). Paclitaxel dose was reduced in four patients (three due to haematological toxicity and one due to neurotoxicity), while pegylated LD dose reduction was performed in 29 patients (20 due to haematological toxicity, seven due to PPE, two due to stomatitis) (*P*< 0.001). Defining as a delay a more than 1 week interval between scheduled treatment day and actual day of treatment administration, there were 12 delays in CP (11 due to haematological toxicity and one due to hospitalization) and 26 delays in CLD (24 due to haematological toxicity, one due to GI toxicity and one due to PPE) (*P *= 0.006).

### Toxicity

A total of 177 patients (90 in CP, 89 in CLD) receiving at least one cycle, were evaluable for the safety analysis. Toxicity data are available for 173 (89 in CP and 84 in CLD). The incidence of selected toxicities and supportive care is summarized in Tables 2 and 3. Haematological toxicity was the most common toxicity in both groups. Grade 3-4 neutropenia did not differ significantly between the groups (30% in CP, 35% in CLD), while severe thrombocytopenia was higher among the CLD patients (11% in CLD versus 2% in CP, *P *= 0.016). Febrile neutropenia occurred in nine patients (four in CP and five in CLD). Few patients discontinued treatment due to haematological events (six due to severe neutropenia in CP, one due to severe thrombocytopenia in CLD). There were no toxic deaths. Patients in the paclitaxel group had significantly more neurotoxicity of any grade and higher incidence of severe neurotoxicity (grade 1-2 neurotoxicity 57% in CP versus 13% in CLD (*P *= 0.003, grade 3-4 neurotoxicity 7% in CP versus 0% in CLD, *P *= 0.029). Five patients in CP discontinued treatment due to neurotoxicity. Hypersensitivity reactions (HSRs) were more common in CP, mostly grade 1-2 (31% in CP versus 7% in CLD) with two cases of grade 3 HSR (one in each group). Five patients discontinued treatment because of an HSR (three in CP and two in CLD). There was only one reported case of HSR due to carboplatin in a patient receiving CP. All other reported cases of HSRs were due to paclitaxel or pegylated LD. Alopecia was significantly more common for patients receiving paclitaxel (grade 2 alopecia 63% in CP versus 6% in CLD, grade 3 alopecia 20% in CP versus 5% in CLD, *P *= 0.003). Incidence of PPE and skin toxicity was higher in CLD (grade 1-2 38% versus 9% in CP, *P *= 0.003) with only one patient developing grade 3 prolonged skin toxicity, because of which treatment was discontinued. There were no significant differences in the rates of hospitalization, antibiotic or G-CSF use between the groups, however, the rate of red blood cell transfusion was higher in CLD (3% in CP versus 14% in CLD, *P *= 0.015).

**Table 2:** Toxicity and supportive care - incidence of severe toxicity among treated patients.

	**Group A (CP)** ***N *= 89**				**Group B (CLD)** ***N *= 84**			
	
	**Grade 1**	**Grade 2**	**Grade 3**	**Grade 4**	**Grade 1**	**Grade 2**	**Grade 3**	**Grade 4**
	
Neutropenia	14 (16)	20 (22)	18 (20)	9 (10)	13 (15)	20 (24)	23 (27)	7 (8)
Anaemia	29 (33)	0 (0)	3 (3)	0 (0)	27 (32)	23 (27)	7 (8)	1 (1)
Leucopenia	24 (27)	23 (26)	5 (6)	1 (1)	25 (30)	30	4 (5)	1 (1)
Thrombocytopenia†	1 (1)	6 (7)	2 (2)	0 (0)	4 (5)	7 (8)	9 (10)	1 (1)
Stomatitis		1 (1)		0 (0)	7 (8)	5 (6)	3 (3)	0 (0)
Nausea/vomiting	18 (20)	10 (11)	1 (1)	0 (0)	16 (19)	12 (14)	4 (5)	0 (0)
Diarrhoea	5 (6)	1 (1)	1 (1)	0 (0)	5 (6)	1 (1)	0 (0)	0 (0)
Infection	1 (1)	3 (3)		0 (0)	3 (4)	1 81)	1 (1)	1 (1)
Neurotoxicity‡	24 (27)	27 (30)	5 (6)	1 (1)	19 (12)	1 (1)	0 (0)	0 (0)
Alopecia‡	1 (1)	56 (63)	18 (20)	0 (0)	12 (14)	5 (6)	4 (5)	0 (0)
Allergy	18 (20)	9 (10)	1 (1)	0 (0)	4 (5)	2 (2)	1 (1)	0 (0)
Skin	6 (7)	2 (2)	0 (0)	0 (0)	9 (11)	12 (14)	1 (1)	0 (0)
Hand and foot	0 (0)	0 (0)	0 (0)	0 (0)	2 (2)	8 (10)	0 (0)	0 (0)
Fatigue	12 (13)	6 (7)	0 (0)	0 (0)	8 (10)	6 (7)	0 (0)	0 (0)
Fever		5 (6)	0 (0)	0 (0)	2 (2)	4 (5)	0 (0)	0 (0)
Anorexia	4 (4)	2 (2)	0 (0)	0 (0)	5 (6)		0 (0)	0 (0)
Cardiac	0 (0)	0 (0)	0 (0)	0 (0)	0 (0)	1 (1)	0 (0)	0 (0)
Arthralgias/myalgias	18 (20)	8 (9)	0 (0)	0 (0)	6 (7)	0 (0)	0 (0)	0 (0)

†Rate of severe thrombocytopenia is higher among patients treated with CLD (group B) (11% versus 2%, p = 0.016)

‡Rates of severe neurotoxicity and alopecia are higher among patients treated in group A (CP; 7% versus 0%, *P *= 0.029 and 20% versus 5%, *P *= 0.003)

**Table 3:** Toxicity and supportive care - supportive treatment.

	CP *N *= 90	CLD N = 87
Antibiotics	9 (10)	14 (16)

Granulocyte colony-stimulating factor	41 (46)	45 (52)

RBC transfusion †	3 (3)	12 (14)

Platelet transfusion	0 (0)	3 (3)

Hospitalization	11 (12)	13 (15)

† Red blood cell (RBC) transfusion rate is higher among patients treated in group B (3% in group A versus 14% in group B, *P *= 0.015)

### Efficacy

**Figure 3:**
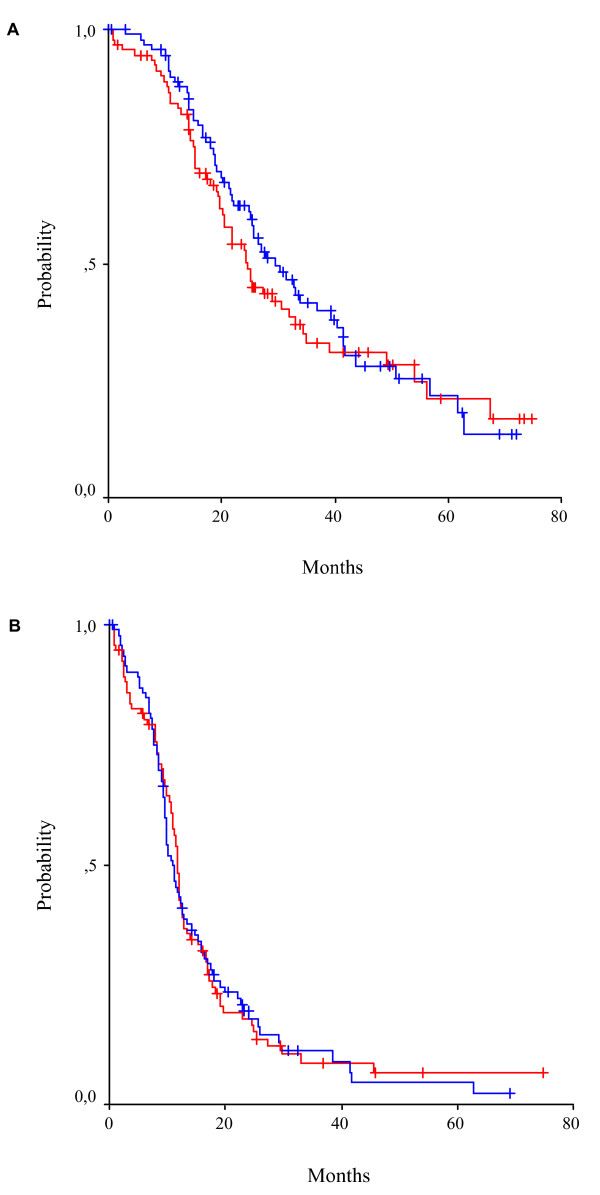
**Kaplan-Meier curves for (A) overall survival, (B) time to progression**. The blue line corresponds to group A, while the red line to group B.

There were 33 Complete Responders (CRs) (34%; 95% CI 25%-45%) and 23 Partial Responders (PRs) (24%; 95% CI 16%-34%) in CP, for an overall response rate (ORR) 57% (95% CI 47%-67%). In CLD there were 21 CRs (23%; 95% CI 15%-32%) and 26 PRs (28%; 95% CI 19%-38%), with a 51% ORR (95% CI 40%-61%).) The difference was not statistically significant. Similarly, when response rates were analysed for measurable disease and only CA125 elevated, there were no statistically significant differences between the groups (Table 4). Median follow-up was 43.6 months (95% CI 0.1-74.8), median TTP was 10.8 months (95% CI 9.2-12.4) in CP and 11.8 months (95% CI 11.2-12.3) in CLD, with no statistical difference (*P *= 0.904). Finally, OS did not differ significantly between the groups, reaching 29.4 months (95% CI 21.9-36.9) in CP and 24.7 months (95% CI 21.0-28.3) in CLD (p = 0.454) (Figure 3).

**Table 4:** Response

	All patients		Measurable disease at trial initiation		Elevated CA125	
	**Group A** N= 96	**Group B** ***N *= 93**	**Group A** N= 79	**Group B** ***N *= 80**	**Group A** N= 17	**Group B** ***N *= 13**

**CR**	33 (34)	21 (23)	24 (30)	17 (21)	9 (53)	4 (31)

**PR**	23 (24)	26 (28)	22 (28)	24 (30)	1 (6)	2 (15)

**OR**	55 (57)	47 (51)	46 (58)	41 (51)	10 (59)	6 (46)

**SD**	16 (17)	15 (16)	15 (19)	14 (18)	1 (6)	1 (8)

**PD**	9 (9)	10 (11)	7 (9)	8 (10)	2 (12)	2 (15)

**Early Tumor death**	0 (0)	3 (3)	0 (0)	3 (4)	0 (0)	0 (0)

**Treatment discontinuation prior to evaluation**	4 (4)	6 (6)	2 (3)	6 (8)	2 (12)	0 (0)

NA*	17 (18)	6 (6)	9 (11)	8 (10)	2 (12)	4 (31)

*NA (not assessed): in case of measurable disease no computed tomography scans after treatment; in case of CA125 elevation no CA125 value after treatment.

There were no significant differences in any of the rates between the two groups; more specifically, the rates of CR (complete response), PR (partial responbse) and OR (overall response) were as follows:

All patients: (*P *= 0.072, 0.619 and 0.309, respectively).

Patients with measurable disease: (*P *= 0.208, 0.861 and 0.427, respectively).

Patients with evaluable disease (elevated CA125 and/or effusions): (*P *= 0.283, 0.565 and 0.713, respectively)

### Prognostic factors

Employing the Cox proportional hazards model, a univariate and multivariate analysis was performed in order to assess the effect of pre-specified prognostic factors on patients' survival (Table 5). Overall, PS = 0 and longer PFI (> 12 months) were important independent prognostic factors for survival in this patient population. PFI was an important independent prognostic factor (PFI 12.1-24 months, *P *= 0.013 in univariate and *P *= 0.009 in multivariate analysis; PFI > 24 months, *P*< 0.001 both in uni- and multi-variate analysis). Performance status (PS) was also an important prognostic factor, with PS = 0 being significantly better than PS 1-2 (*P *= 0.001 in uni- and *P *= 0.003 in multi-variate analysis).

**Table 5:** Prognostic factors. Cox regression analysis for patients' survival.

	Univariate			Multivariate		
	**HR**	**95% CI**	***P***	**HR**	**95% CI**	***P***

**Age**						
< = 65	1	-	-			
> 65	0.83	0.57-1.21	0.329			
**Performance status**						
0	1	-	-	1	-	-
1-2	1.96	1.32-2.90	0.001	1.89	1.25-2.88	0.003
**Previous exposure to taxanes**						
No	1	-	-			
Yes	1.18	0.62-2.27	0.610			
**Disease status**						
Non-measurable	1	-	-			
Measurable	1.49	0.88-2.55	0.141			
**Platinum-free interval**						
6-12 months	1	-	-	1	-	-
12.1-24 months	0.58	0.37-0.89	0.013	0.54	0.34-0.86	0.009
> 24 months	0.37	0.22-0.61	< 0.001	0.36	0.21-0.61	< 0.001
**Randomization group**						
A	1	-	-	1	-	-
B	1.15	0.78-1.66	0.455	1.19	0.80-1.76	0.399

CI, confidence interval; HR, (Hazard Ratio)

### Post-progression therapy

A total of 159 relapses (or disease progressions) were recorded (81 in CP and 78 in CLD). Detailed information on post-progression therapy was available for most patients: 122 patients received further chemotherapy (61 in CP, 61 in CLD), 29 patients did not receive post-progression therapy (16 in CP and 13 in CLD) and information was not available for eight patients (four in CP and four in CLD). No details on possible surgical treatment on relapse were available. Twenty-four patients (39%) in CP versus 21 (34%) in CLD received a platinum combination again. One patient in CP received paclitaxel again versus nine in CLD, while docetaxel was administered in five patients in CP and seven in CLD (9% in CP versus 26% in CLD received taxane again). A total of 26 patients (42%) in CP received pegylated LD as post-progression treatment versus four patients (6%) in CLD who received LD again.

## Discussion

The combination of carboplatin-paclitaxel is a global standard following recent consensus recommendations [20,4]. Although this treatment is highly effective, most patients recur. The majority are platinum-sensitive at first relapse, thus, candidates for re-treatment with platinum. Indeed, these patients will be generally re-treated with a platinum-taxane combination, especially in the light of recent trials showing advantage over platinum monotherapy [9]. However, the cumulative neurotoxicity of both drugs, as well as the increased risk of neurotoxicity for patients in relapse and the further experience of alopecia, are essential considerations when selecting second-line therapy [21]. As treatment at relapse is rarely curative, toxicity, tolerance, ease of administration and QoL should be interrelated to efficacy and survival prolongation when novel platinum-based combinations are evaluated for patients with platinum-sensitive OC.

This study was originally designed in 1999, in an era when information was totally lacking regarding the ideal regimen for platinum-sensitive recurrent OC. This is still a question that has not been clearly and conclusively answered today. The large ICON-4 study (paclitaxel-platinum versus platinum) was then ongoing and was published in 2003. Therefore, both groups in this study were 'experimental' at the time of design, with preliminary evidence for the one that proved to be the standard (paclitaxel-platinum). At the time it was decided to conduct a randomized phase II study, anticipating the accrual, and also to investigate whether either of the two regimens would be worth exploring further in larger studies [22]. We present the results of this evaluation of pegylated LD plus carboplatin and the standard combination of paclitaxel plus carboplatin in platinum-sensitive OC patients. This study, with the limitations of a randomized phase II design, demonstrates that the combination of pegylated LD plus carboplatin as second-line treatment of platinum-sensitive OC patients is feasible, well-tolerated and effective and shows a different toxicity profile, with significantly less neurotoxicity and alopecia than the standard regimen of paclitaxel plus carboplatin.

Pegylated LD is a unique formulation of doxorubicin, where encapsulation in liposomes confers different pharmacokinetic characteristics and a more favourable toxicity profile compared to conventional doxorubicin [23]. In phase II and III second-line monotherapy studies, pegylated LD is equally effective to paclitaxel and superior to topotecan, regardless of platinum sensitivity [12,13]. A dose of 40 mg/m^2 ^every 28 days seems the most commonly utilized in clinical practice today, being effective and well tolerated in patients with advanced ovarian cancer [24]. In recent phase II studies different schedules and dosage regimens of pegylated LD plus carboplatin have been assessed [14-16]. In most published studies the pegylated LD dose is lower than the one used in our study, ranging from 30-40 mg/m^2 ^pegylated LD combined with carboplatin AUC 5-6, administered every 28 days, except in the MITO-2 first-line study were carboplatin AUC 5 was combined with pegylated LD 30 mg/m^2 ^and was administered every 21 days [25,26]. The dose of 45 mg/m^2 ^in our study is higher. Information regarding the optimal dose of pegylated LD in combinations and, in particular with carboplatin was limited at the time of initiation of our study and included data from phase I and II studies, were the dose of pegylated LD ranged from 30 - 50 mg/m^2 ^every 4 weeks (dose intensity, 10-12.5 mg/m^2 ^weekly) combined with carboplatin AUC5 [26], while the licenced dose for pegylated LD as monotherapy was 50 mg/m^2 ^every 4 weeks. We chose the dose of 45 mg/m^2 ^every 4 weeks in an attempt to maintain reasonable dose intensity based on available information at the time. Today it is known from retrospective analyses and prospective studies that the lower dose intensity can achieve equally effective results with better tolerance [26]. The higher dose utilized in our study was well tolerated in our patients and haematological toxicity was similar to, or even lower than, other reports such as the recently presented rates of neutropenia in the CALYPSO trial where an even lower dose of CLD was used (30 mg/m^2 ^every 4 weeks) [27,28]. This inconsistency could possibly be explained by the fact that blood counts were only routinely performed at day 1 in our study and only in cases of fever > 38°C during cycles. Therefore, neutropenic events might have been missed, resulting in some underestimation of haematological toxicity. Furthermore, we note that, as per study protocol, if ANC was < 1.5 × 10^9^/L on day 1, GCSF was administered in order to speed recovery. The above might also explain the relatively low rate of grade 4 neutropenia in our study (10%) and the apparent discrepancy with the rate of GCSF used (50%). Rates of severe anaemia and fatigue were also somewhat lower than previously reported, although anaemia of any grade was more common in the CLD arm and transfusion rates were significantly higher in the CLD arm; information on erythropoietin use was not available.

Expectedly, the only non-haematological toxicity higher in the CLD arm was skin toxicity and PPE. This was, however, mostly mild to moderate, resolving with short treatment delays and only in one case grade 3 toxicity caused treatment discontinuation. On the other hand the paclitaxel-carboplatin combination induced significantly higher rates of neurotoxicity causing treatment discontinuation in five cases, while alopecia and hypersensitivity reactions were also significantly more common in CP. Similarly, the rates of hypersensitivity reactions in the CALYPSO trial reported recently at ASCO 2009 [28], were significantly higher in the CP arm (18% versus 5%). However, noticeably higher rates of HSRs related to carboplatin were reported in the CALYPSO trial, again in the CP group (10% grade 2, 9% grade 3). In our study only in one case the hypersensitivity reaction was due to carboplatin (in a CP patient); in all other reported cases the HSR was due to either paclitaxel in the CP group or pegylated LD in the CLD group. We utilized standard premedication in both groups (orally [12 h] and intravenously [30 min] prior to paclitaxel infusion, 30 min intravenously prior to pegylated LD infusion). It is also important to note that overall the rate of discontinuation due to toxicity was significantly higher in the paclitaxel group (13.5% versus 3%, *P *= 0.016), as was seen in the CALYPSO results (toxicity-related early treatment discontinuation in CP 15% versus 7% in CD) [28]. With regards to neurotoxicity, the rate of grade 2-4 neurotoxicity seen in our study (37%) with CP was higher than that reported in the ICON4 trial (20%) [9]. This could be explained by the fact that most patients in our study had previously received a platinum-taxane combination (90% total, 88% in CP), while in the ICON4 more than half (57%) had not received a taxane at first-line treatment. We, therefore, believe that our study reflects the problem of neurotoxicity more accurately in the context of the recent changes of first-line treatment in ovarian cancer. Similarly, the CALYPSO trial included patients pretreated with both a platinum and a taxane, as this is nowadays common practice in most parts of the world in first-line treatment of advanced ovarian cancer. In their recently presented results, the CALYPSO investigators showed a significantly higher severe neuropathy rate in the CP arm (27% versus 4%) and concluded that the combination of PLD-carboplatin was well tolerated with lower rates of severe and long-lasting (neuropathy) toxicities [28]. Although QoL measurements were not undertaken in our study, the CLD toxicity profile, with less neurotoxicity and alopecia, seems more favourable. However, this should be conclusively investigated in larger randomized studies that incorporate patient perception measurements. Such QoL measurements were undertaken in the large CALYPSO trial, the results, however, have not yet been presented.

Our study was not powered to detect differences in survival; therefore, data on TTP and OS are only indicative. The observed response rates in our study, as well as our data on TTP and OS are comparable to those reported in the two large randomized phase III trials on platinum-sensitive disease [9,11]. They come to further underline the efficacy of platinum-based combinations in a population characterized (65% of patients) of predominantly long PFI (> 12 months). Additionally, in accordance to previous evidence, our data demonstrated that longer PFI and better PS are significant independent prognostic factors for survival [7]. The above compare favourably with recently presented results both in first- and second-line treatment of advanced OC patients: the MITO-2 study, a large randomized comparison of CP versus CLD as first-line treatment showed equal response rate and favourable toxicity profile for the pegylated LD combination compared to the standard paclitaxel combination while PFS analysis is still pending [29]. Furthermore, the CALYPSO trial in the second-line setting as our study is, showed significant superiority of the pegylated LD combination in terms of tolerance and PFS (18% reduction in risk of recurrence; HSR 0.82; *P *= 0.005) [28].

## Conclusions

Our data support the clinical efficacy and tolerability of pegylated LD combined with carboplatin for the second-line treatment of platinum-sensitive OC patients. The CALYPSO phase III study evaluated a similar combination of CLD versus CP, using lower dose intensity for pegylated LD. The recently presented results of this large multinational study come to strengthen, with randomized evidence, the previous indications of a more favourable toxicity profile of pegylated LD combined with carboplatin compared to a paclitaxel combination [24] in the second-line setting. The results of our study, regarding the safety and toxicity of the CLD combination, are consistent with and add to the evidence of the CALYPSO trial. The combination of pegylated LD plus carboplatin has satisfactory activity and shows a different toxicity profile to the standard combination of paclitaxel-carboplatin, with significantly less neuropathy and alopecia, worthy of further evaluation and use as an alternative evidence-based treatment option for patients with platinum-sensitive OC.

## Abbreviations

ANC: absolute neutrophil count; AUC: area under the curve; CBC: complete blood count; CI: confidence interval; CLD: carboplatin plus pegylated LD; CP: carboplatin plus paclitaxel; G-CSF: granulocyte colony-stimulating factor; HSR: hypersensitivity reactions; LD: liposomal doxorubicin; OC: ovarian cancer; ORR: overall survival rate; OS: overall survival; PFI: platinum-free interval; PFS: progression-free survival; PPE: Palmer-planter erythrodysthesia; PS: platinum sensitivity; QoL: quality of life; RR: response rate; TTP: time to progression.

## Competing interests

The authors declare that they have no competing interests.

## Authors' contributions

All authors contributed substantially to the conception and contact of the study, data acquisition and analysis and they all read and approved the final manuscript. DB, GA, CP and AMD primarily contributed to the conception, design and coordination of the study and data acquisition, HL, DB, CP, AB, GF and AMD have also been involved in drafting the manuscript and revising it critically, while HPK, PK, ET, TM, ES, EB, CC, PP and DP participated in the contact of the study and data acquisition and analysis.

## Pre-publication history

The pre-publication history for this paper can be accessed here:

http://www.biomedcentral.com/1741-7015/8/3/prepub
